# Has the time to come leave the “watch-and-wait” strategy in newly diagnosed asymptomatic follicular lymphoma patients?

**DOI:** 10.1186/1471-2407-12-210

**Published:** 2012-05-31

**Authors:** Antonio Rueda, María Casanova, Maximino Redondo, Elisabeth Pérez-Ruiz, Ángeles Medina-Pérez

**Affiliations:** 1 Department of Oncology, Hospital Costa del Sol, Marbella, Spain; 2 Biochemistry, Hospital Costa del Sol, Marbella, Spain

## Abstract

**Background:**

Historically, the median overall survival for follicular lymphoma (FL) has been considered to be 9-10 years, and no treatment had ever prolonged this time period. Studies conducted more than 20 years ago demonstrated that treating patients with asymptomatic FL at the onset of the disease did not increase their survival, and that almost 20% of these patients did not need any treatment in the first 10 years of follow-up. Based on these facts, most clinical practice guidelines recommend active surveillance policies for patients with asymptomatic FL.

**Discussion:**

The introduction of antiCD-20 monoclonal antibodies, over the last 15 years, has significantly increased the median survival rate to above 14 years. This improvement was achieved before the combination of rituximab and chemotherapy regimens became extensively used in patients with symptomatic disease. Therefore, this increase in survival may currently be more significant. At present, several clinical trials have evaluated low-toxicity therapies that prolong progression-free periods, among which rituximab monotherapy, radioimmunotherapy or the combination of rituximab with bendamustine are the most relevant. Unfortunately, these clinical trials have included only patients with symptomatic FL. The results of a recently reported clinical trial show that treatment with single-agent rituximab prolongs progression-free survival rates, time to new treatment and the quality of life of asymptomatic patients, as compared with the active surveillance strategy. Longer follow-up of these results and data regarding overall survival are awaited before this treatment can be recommended as the standard initial therapy.

**Summary:**

There are different therapeutic possibilities for asymptomatic FL patients, but no data are currently available to indicate which option is the best. Patients need to understand the risks and benefits of observation versus treatment before a final decision can be made. For patients who want active treatment the administration of four weekly rituximab doses should be considered.

## Background

Follicular lymphoma (FL) is the second most common subtype of lymphoma in Western Europe, as it represents 22-25% of non-Hodgkin´s lymphomas (NHL), according to the WHO histological classification [1,2]. The annual incidence of FL increased from two to three per 100,000 persons/year during the 1950s to five to seven per 100,000 persons/year in 2009, with a prevalence of 40 cases per 100,000 people [3].

FL originates in follicular-centre B cells, usually in the lymph nodes, and maintains the phenotypic characteristics and gene expression of these cells. Approximately 85% of FL patients present with chromosomal translocation t(14;18), which leads to overexpression of the BCL-2 oncogene and results in resistance to apoptosis. t(14,18) is not specific for FL, as it is present in 30% of diffuse large B-cell lymphomas (DLBCL) and even in some healthy individuals [4].

Morphologically, FL is composed of small B-cells (centrocytes) and large B-cells (centroblasts) that are clonally related. The morphological distribution follows a nodal growth pattern similar to that of the germinal centres observed in secondary lymphoid follicles. Neoplastic follicles may be prevalent in tumoural tissue; the rest of the tumour exhibits a diffuse growth pattern [1]. FL is classified into three grades according to the number of centroblasts present in the tumour tissue (Table 1). In grades 1 and 2, centrocytes prevail; in grade 3 large cells are more numerous. Grade 3 is divided into 3A (when centrocytes are still present) and 3B (characterised by the presence of solid sheets of centroblasts). Although grade 3B FL has a worse prognosis and its natural history and treatment are similar to those of DLBCL, the genetic characteristics and clinical behaviour suggest that grade 3A FL exhibits indolent behaviour very similar to that of grade 1-2 FL [5].

**Table 1:** **Follicular lymphoma grading according to the WHO classification**^**1**^

**Grading Definition**	
Grade 1–2 (low grade) 0–15 centroblasts per hpf*	
· 1 0–5 centroblasts per hpf	
· 2 6–15 centroblasts per hpf	
***Grade 3 >15 centroblasts per hpf***	
· 3A: centrocytes present	
· 3B: solid sheets of centroblasts	
**Follicular Pattern**	
· Follicular >75%	
· Follicular and diffuse 25–75%	
· Focally follicular <25%	
· Diffuse 0%**	

* hpf = high-power field of 0.159 mm^2^.

** Diffuse areas containing >15 centroblasts per hpf are reported as DLBCL with FL (grades 1 to 2, 3A or 3B). Note that in small biopsies the absence of follicles may reflect a sampling error.

FL is the most common indolent lymphoma accounting for 70% of all indolent lymphomas. The treatment used for FL has been generally accepted for other less frequent indolent lymphomas. Usually the first symptom is the asymptomatic presence of enlarged peripheral nodes in the neck, and in the axillary or inguinal areas.

Occasionally, adenopathies increase and decrease in size for prolonged intervals before diagnosis. Some patients present a bulky abdominal mass not associated with urinary/intestinal obstruction. Although patients only present with one or two node areas that are clinically affected, the staging study showed dissemination affecting the spleen, liver or bone marrow in 40%, 50% and over 60% of patients, respectively. Extranodal involvement is infrequent [6].

Once a diagnosis of FL is confirmed, the pretreatment evaluation determines the extent of the disease and provides information about the individual's comorbidities that are likely to have an impact on treatment options. Of particular importance is the information gathered from this evaluation, as it is used to determine the patient's Follicular Lymphoma International Prognostic Index (FLIPI) [7] and FLIPI 2 [8] scores, which determines whether the patient is at risk for progression (Table 2) [7,8].

**Table 2:** Follicular Lymphoma International Prognostic Index (FLIPI) and Follicular Lymphoma International Prognostic Index 2 (FLIPI 2)

**FLIPI**				
***Risk factors***				
· Age <60 years vs. ≥60 years				
· Hemoglobin ≥12 g/dL vs. <12 g/dL				
· Serum LDH ≤ ULN vs. >ULN*				
· Ann Arbor stage I-II vs. III-IV				
· No. of nodal sites ≤4 vs. >4				
***Risk group***	***No. of factors***	5–year OS**	10–year OS**	***Relative risk***
Good	0–1	91%	71%	1
Intermediate	2	78%	51%	2.3
Poor	≥3	53%	36%	4.3
**FLIPI 2**				
***Risk factors***				
· Beta-2-microglobulin > ULN*				
· Longest diameter of the largest involved node > 6 cm				
· Bone marrow involvement				
· Hemoglobin level < 12 gr/L				
· Age older than 60 years				
***Risk group***	***No. of factors***	3-year PFS***	5-year PFS***	***Relative risk***
Low	0	90.9%	79.5%	1
Intermediate	1-2	69.3%	51.2%	3.19
High	3-5	51.3%	18.8%	5.76

* ULN: under limits normal.

** OS: overall survival.

*** PFS: progression free survival.

Most patients are diagnosed with FL when they have an advanced stage of the disease (less than 20% present with stages I or II), although a significant percentage of patients just present with an enlarged node noted by palpation (asymptomatic FL).

FL is characterised by a chronic remission/relapse pattern. FL natural history is characterised by alternating periods of treatment, achievement of a partial/total remission, and periods free of disease and relapse or progression of the lymphoma, which requires additional treatment. With each new treatment remissions are less frequent and progression-free periods are shorter. Occasionally, partial and short spontaneous remissions can occur. Finally, patients die after developing refractory FL, FL transformation to a more aggressive lymphoma (10-70% of cases), acute toxicity, or of causes unrelated to the disease. However, FL is not necessarily an incurable disease. A randomized study with a long follow-up period, conducted in the pre-rituximab era, showed that about 15-20% of patients never relapse or die without known disease at nearly 20 years after treatment [9].

With the treatments that were applied 10-15 years ago in the so-called pre-rituximab era, the median survival ranged from 9 to 10 years and no treatment was shown to increase overall survival [10]. The aim of the treatment was to improve patients’ quality of life. Therefore, asymptomatic patients were not treated until the disease was progressive (watch-and-wait strategy). Thus, patients were spared the acute and late toxic effects of the treatment.

In the past 15 years, the introduction of rituximab into the treatment of patients with FL has resulted in a significant change in the diseases’ prognosis and natural history. The median survival has increased to approximately 14 years [11], and progression-free survival periods without treatment have been extended to nearly 5 years [12]. All this has been achieved with just a slight increase in toxicity.

Unfortunately, advanced-stage FL is still incurable for the majority of patients. Thus, current therapies are aimed at achieving optimal palliation by inducing long periods of durable remissions with assumable acute and late toxicity, which limit the potential development of shared "cross-resistance" to other therapies in the future [13].

In this new setting and in the light of recent evidence, application of the “watch and wait” policy to asymptomatic patients should be reviewed.

## Discussion

### Definition of asymptomatic follicular lymphoma

There is not a universally accepted definition with which to identify a LF patient as being asymptomatic. The definitions available were provided in retrospective studies by cooperative groups that sought to describe clinical risk factors for disease progression. At present, the most common criteria for identifying low-risk/low-tumour burden asymptomatic patients are those established by the Groupe d´Etude des Lymphomes Folliculares (GELF) [14,15]. According to this group, low-risk/low-tumour burden asymptomatic patients who are likely to remain untreated were those who did not meeting any of the following criteria:

• Lymph node or extranodal mass with a diameter > 7 cm

• Involvement of at least three nodal regions, each with a diameter > 3 cm

• Systemic symptoms/B-symptoms

• Splenomegaly below the umbilicus

• Compression syndrome (ureter, intestinal, orbital, and others)

• Serous pleural/peritoneal effusion (regardless of cell count)

• Leukemic expression with circulating tumour cell count > 5.0 x 10^9^ x L

• Cytopenias (concentration of granulocytes < 1.0 x 10^9^ x L and platelet count < 100 x 10^9^ x L)

These are the criteria employed in most clinical trials and recommended by the Clinical Guidelines for the Treatment of Follicular Lymphoma of the American National Comprehensive Cancer Network (NCCN) [16].

### Clinical trials supporting the “watch-and-wait” strategy

Three randomized trials assessed the effectiveness of active surveillance without first-line treatment of advanced FL. The American National Cancer Institute (NCI) randomized 89 patients with advanced indolent lymphoma to “watch and wait” or to undergo aggressive combined modality treatment with prednisone, methotrexate, adriamycin, cyclophosphamide, etoposide, mechlorethamine, vincristine and procarbazine (ProMACE-MOPP), followed by total nodal irradiation (TNI) [17]. Survival in both groups at 5 years was over 75%, but progression-free survival was 51% in the chemotherapy group and 12% in the watchful waiting group.

In France, the GELF group randomized patients with advanced FL and low-tumour-burden to the watch-and-wait strategy, or to treatment with prednimustine or interferon alfa [14]. Overall survival at 5 years was similar for the three arms of the study.

The most significant study was that conducted by the British National Lymphoma Investigation Group, which randomized 309 patients with advanced indolent lymphoma (204 with FL) to immediate treatment with chlorambucil or to systemic therapy deferred until disease progression [18]. In both arms modest doses of palliative radiotherapy for the treatment of symptomatic adenopathies were permitted. With a 16-year median follow-up, the overall survival time was similar between both groups. Ten years following the initiation of the study, 19% of the patients treated with the watch and wait approach had still not required systemic therapy (40% of patients older than 70 years). However, it should be noted that the patients on immediate systemic treatment had higher response rates (63% of complete remission) than the patients who were initially untreated (27% of complete remission).

These three randomized studies revealed that 60-75% of the patients treated with the watchful-waiting protocol required treatment during the first 2-3 years of follow-up.

The situation in stage I-II follicular lymphoma, according to the Ann Arbor staging index, differs. The administration of radiotherapy (24-36 Gy) to involved nodal areas resulted in progression in 50% of the patients at 10 years [19,20]. However, when high morbidity is expected due to radiotherapy, or when the patient refuses to receive radiotherapy, watchful waiting can be a reasonable strategy. Two retrospective studies have shown that this strategy does not affect overall survival (85% at 10 years) and, with a median follow-up period of 7.2 and 6.3 years, progression was not observed in 63% and 50% of the patients, respectively, so therapy was not required [21,22].

### Active surveillance or watch and wait: Advantages and disadvantages

The watch-and-wait policy applied to asymptomatic FL patients involves more intensive surveillance than in patients in remission after first-line treatment. This is the reason why it is named “active surveillance” (or “watchful waiting”). Anticancer treatment is not administered, but surveillance visits are more frequent (every 3 months) in order to detect symptomatic progression of the disease and initiate treatment before the onset of further complications.

The advantages and disadvantages of deferring anticancer treatment in asymptomatic FL patients in the prerituximab era are shown in Table 3. The effectiveness of FL therapies has improved in recent years. Long remission periods are now achieved and, in certain cases, by treatments involving low acute toxicity risk. Therefore, nowadays the main disadvantage of undertaking active surveillance for indolent FL is that a treatment with a very favourable therapeutic index (efficacy/toxicity) is delayed.

**Table 3:** Advantages and disadvantages of deferring treatment in asymptomatic FL patients (watchful waiting)

**Advantages**	
· Patients are spared the effects of acute toxicity	
· Patients are spared the effects of serious late toxicity (i.e., myelodysplasia)	
· The development of shared "cross-resistance" to other therapies is avoided	
**Disdvantages**	
· Risk of severe complications associated with the disease during the interval between visits	
· The chances of obtaining a good antitumor response with the first course of treatment can decrease [16]	
· The risk of FL transformation into aggressive lymphoma can increase [23]	
· Anxiety caused by the fact of suffering an oncologic disease that is not being treated	

### What has changed in the rituximab era?

In 1997, rituximab was approved for the treatment of refractory/relapsed FL. Since then, this anti-CD-20 monoclonal antibody has proven to be effective when used as a monotherapy or in combination with chemotherapy at different stages in the FL natural history. Radioimmunotherapy and other antineoplastic agents have also demonstrated significant antitumor activity. The introduction of these drugs has modified some of the arguments on which active surveillance – as a treatment strategy in asymptomatic patients – is based.

### Overall survival has increased

Historically, the median overall survival for FL was considered to be 9-10 years, and no treatment had ever prolonged this time period [10]. However, the introduction of anti CD-20 monoclonal antibodies has significantly increased the median survival rate to above 14 years [11]. This improvement was detected before the combination of rituximab and chemotherapy regimens was extensively employed as an early treatment, and before the use of monotherapy with rituximab as a maintenance therapy after induction treatment. Both strategies have been shown to increase survival rates since 2003 [24,25]. Therefore, this increase in survival may currently be more significant.

### First-line treatment with immunochemotherapy may prolong progression-free periods and the time to the next treatment

The benefit of adding rituximab to combination chemotherapy (immunochemotherapy) has been demonstrated in several randomized trials of chemotherapy with or without rituximab [26-30]. All of these trials have demonstrated improved response rates and time to progression when rituximab was added; many also showed an improvement in overall survival with rituximab. Progression-free survival rates at 3 years were increased by approximately 15 to 30%.

An international multicenter trial randomly assigned 321 patients with previously untreated stage III/IV FL to receive eight cycles of CVP (cyclophosphamide, vincristine and prednisone) or CVP plus rituximab (R-CVP) [26]. The patients assigned to R-CVP demonstrated significantly higher rates of overall (81% versus 57%) and complete (41% versus 10%) responses. At a median follow-up period of 30 months, R-CVP improved median time to progression (32 months versus 15 months). At a median follow-up period of 53 months, the patients who received R-CVP had significantly higher rates of 4-year overall survival (83% versus 77%) [27].

In a second randomized trial, 428 patients with untreated advanced-stage FL were randomly assigned to treatment with CHOP (cyclophosphamide, doxorubicin, vincristine and prednisone) with or without rituximab [28]. The patients assigned to R-CHOP had significantly superior 2-year progression-free survival rates (approximately 85% versus 65%) and overall survival rates (95% versus 90%). Another trial randomly assigned 201 patients with previously untreated stage III/IV FL to receive eight cycles of MCP (mitoxantrone, chlorambucil and prednisone) or MCP with rituximab (R-MCP) [29]. All patients who achieved a complete or partial response were treated with interferon maintenance until relapse. Overall (92% versus 75%) and complete (50% versus 25%) response rates were higher in the R-MCP treatment arm. At a median follow-up of 47 months, the patients assigned to R-MCP demonstrated superior median progression-free (not reached versus 29 months) and 4-year overall survival rates (87% versus 74%).

In a French prospective trial, 185 patients with previously untreated advanced-stage FL were randomly assigned to treatment with chemotherapy (cyclophosphamide, doxorubicin, etoposide, prednisone, plus interferon) with or without rituximab [30]. After a median follow-up of 5 years, the patients assigned to chemotherapy plus rituximab group had significantly higher rates of complete response (67% versus 50%) and longer event-free survival (53% versus 37%) than those who were assigned to the chemotherapy without rituximab group.

### Low-toxicity treatments can prolong progression-free survival

Initial treatment with single-agent rituximab appears promising given its low toxicity profile and good response rates. However, long-term follow-up of such an approach is limited. The following phase-II trials evaluated rituximab (375 mg/m^2^ IV per week for a minimum of 4 consecutive weeks) as initial therapy in patients with FL.

In a prospective trial of single-agent rituximab in 62 chemotherapy-naive patients, most of whom had stage III or IV disease, overall response rates at 6 weeks and at maximum response were 47% and 73%, respectively, with 7% and 37% complete remissions, respectively [31]. At a median follow-up period of 30 months, median progression-free survival was 34 months. Toxicity was mostly infusion related and of brief duration.

A second trial evaluated 50 patients with stage II to IV FL and low tumour burden (no nodal or extranodal mass > 7 cm, B symptoms, splenomegaly, pleural effusion, ascites, organ compression, and elevated serum lactate dehydrogenase or beta-2-microglobulin) [32]. The overall response rate at 50 days was 73%. The median time to progression was approximately 13 months. Toxicity was minimal; the number and severity of adverse events decreased with subsequent infusions.

The North Central Cancer Treatment Group enrolled 37 patients with untreated follicular grade 1 NHL and measurable stage III/IV disease [33]. Patients received intravenous rituximab at a dose of 375 mg/m^2^ four times weekly and no maintenance therapy was provided. The overall response rate was 72%, with 36% complete remission. The median time to progression was 2.2 years and fourteen (39%) patients remained in unmaintained remission with a median follow-up of 2.6 years.

An international trial of 202 patients with previously untreated or relapsed/refractory FL involved the administration of rituximab four times weekly [34]. The 151 patients with responding or stable disease at week 12 were randomised to no further treatment or prolonged rituximab maintenance every 2 months for four doses. At a median follow-up of 35 months, patients who received the prolonged rituximab maintenance treatment had a significantly longer median event-free-survival (23 months versus 12 months) when compared with those randomised to observation with no apparent increase in toxicity. This benefit appears to be maintained during long-term follow-up with 35% of the responders still in remission at 8 years after treatment [35]. Of note, 45% of the newly diagnosed patients in this study treated with extended-schedule rituximab were in remission at 8 years.

Anti-CD20 radioimmunoconjugates, for example ibritumomab tiuxetan and tositumomab, have demonstrated efficacy in relapsed or refractory FL. One prospective trial investigated the use of single-agent tositumomab in patients with selected low tumour burden who had had previously untreated FL. A single course of treatment with tositumomab was given to 76 previously untreated patients with FL, and resulted in overall and complete response rates of 95% and 75%, respectively, and 5-year overall survival and progression free survival rates of 89% and 59%, respectively [36].

Bendamustine plus rituximab (B-R) is a new immunochemotherapeutic regimen with high activity and low toxicity in relapsed and refractory FL [37] that is being evaluated as a first-line therapy. In 513 patients with untreated advanced follicular, indolent and mantle-cell lymphoma, bendamustine (90 mg/m^2^ on days 1 and 2) plus rituximab (375 mg/m^2^ on day 1) given every 28 days for six cycles was compared with standard R-CHOP for six cycles; it also achieved statistically superior progression-free survival (54.8 months versus 34.8 months) and event-free survival (54 months versus 31 months) with less toxicity, including lower rates of grade-3 and -4 neutropenia (10.7% versus 46.5%) and leukocytopaenia (12.1% versus 38.2%). Alopecia did not occur, and neuropathy was very uncommon in the B-R treated patients. There was no difference in overall survival at a median follow-up period of 32 months [38].

### Randomized clinical trials with rituximab in asymptomatic follicular lymphoma

The large amount of information that has been generated over the last 15 years on the treatment of LF has not changed clinical opinion regarding the implementation of active surveillance in the treatment of asymptomatic LF. So far, clinical trials have only included either patients with symptomatic FL or mixed symptomatic and asymptomatic patients without previous stratification that would allow for separate analysis of the data obtained. In some phase II trials – such as those conducted with single-agent rituximab alone [32] or with tositumomab [36] – patients were selected with low-tumour burden using different criteria from those used in the GELF group.

The results of two randomized clinical trials including only asymptomatic LF patients conducted in the rituximab era have recently been reported. The British National Cancer Research Institute Lymphoma Group studied the effect of a low-toxicity therapy proven to be effective on asymptomatic FL patients. Their goal was to assess whether single-agent rituximab therapy significantly delayed the initiation of chemotherapy or radiotherapy, as compared with active surveillance in advanced asymptomatic FL patients according to the GELF groups’ criteria [39]. A total of 463 patients were randomised to three treatment arms:

· Active surveillance with regular visits every 2 months

· Rituximab at 375 mg/m^2^/week for 4 weeks

· Rituximab at 375 mg/m^2^/week for 4 weeks followed by rituximab maintenance at 375 mg/m^2^ every 2 months for two years

During the follow-up period, painful peripheral adenopathies were not treated with radiotherapy, and the criteria for initiating a new anti-lymphoma therapy were established in the protocol. Three quarters of the patients recruited had low/intermediate risk (according to the FLIPI index). Regarding toxicity, only 10 grade 3-4 toxicity events were reported (half were infections) among the 276 patients treated with rituximab (3.5%).

Table 4 shows the response rate for each group. With a median follow-up of 32 months, 44% of the patients in the active surveillance group had initiated anti-lymphoma treatment, while the rate for the patients receiving rituximab alone was 23%; this rate was 10% for the patients treated with rituximab plus maintenance therapy. Table 5 shows disease-free survival rates and the time to next antilymphoma treatment for the three trial arms. So far, no difference has been detected in the overall survival rate (95% at 3 years). However, the follow-up period was too short to assess this parameter.

**Table 4:** Responses in the three arms of the rituximab versus “watch-and-wait” study

**Time of response evaluation**	**7 months**	**13 months**	**25 months**
**Arm A (W&W)**			
+ CRu	3 (2%)	6 (3%)	5 (4%)
PR	6 (3%)	7 (4%)	5 (4%)
ORR	9 (5%)	13 (7%)	10 (8%)
**Arm B (Rx4)**			
CR + CRu	35 (43%)	36 (44%)	30 (40%)
PR	25 (30%)	22 (27%)	10 (13%)
ORR	60 (73%)	58 (71%)	40 (53%)
**Arm C (Rx4RM)**			
CR + CRu	100 (54%)	122 (67%)	98 (70%)
PR	61 (33%)	36 (20%)	12 (9%)
ORR	161 (87%)	158 (87%)	110 (79%)
p value (Arm B vs Arm C)	p = 0.0216	p = 0.0077	p < 0.0001

**W&W:** watch and wait; **Rx4:** rituximab four weekly doses; **Rx4 + RM:** rituximab four weekly doses plus rituximab maintenance; **CR:** complete response; **CRu:** undetermined complete response; **PR:** partial response; **ORR:** overall response rate.

**Table 5:** Progression-free survival and time to next antilymphoma treatment in the rituximab versus “watch-and-wait” study

	**PNRNT 3 years**	**Differences between arms**	**HR p value**	**PFS 3 years**	**Differences between arms**	**HR p value**
W&W	48%	Rx4 vs W&W	0.37 p < 0.001	33%	Rx4 vs W&W	0.46 p < 0.001
Rx4	80%	Rx4 + RM vs W&W	0.20 p < 0.001	60%	Rx4 + RM vs W&W	0.21 p < 0.001
Rx4 + RM	91%	Rx4 + RM vs Rx4	0.57 p = 0.10	81%	Rx4 + RM vs Rx4	0.43 p < 0.001

**PNRNT:** patients not receiving next therapy; **PFS:** progression free survival; **HR:** hazard ratio **; W&W:** watch and wait; **Rx4**: four weekly doses of rituximab; **Rx4 + RM**: four weekly doses of rituximab plus rituximab maintenance.

Preliminary results from a quality of life (QOL) analysis of this trial showed that the patients who received treatment with rituximab had reduced anxiety and improved functional well-being [40]. At baseline, patient QOL scores were similar or superior to those of the general population (as assessed by the FACT-G questionnaire), with the exception of inferior emotional well-being. Although the patients treated with the “watch and wait” protocol also reported improvements in some QOL parameters, the greatest improvements in emotional and functional well-being were observed in the patients who received either rituximab maintenance therapy or monotherapy [40].

The Eastern Cooperative Oncology Group have reported the E4402 randomized phase III study (RESORT) comparing two different rituximab dosing strategies for low tumour burden FL (according to the GELF criteria) [41]. Patients received rituximab at 375 mg/m^2^ weekly for four doses and responders were randomized to rituximab maintenance (MR) (single dose rituximab every 3 months) or rituximab retreatment (RR) (rituximab four times weekly at disease progression). Each strategy was continued until treatment failure. The primary endpoint, time to treatment failure (TTTF), was defined as progression within 6 months of last rituximab dose, no response to rituximab retreatment, initiation of alternative therapy or inability to complete protocol therapy. Secondary endpoints included time to first cytotoxic therapy, quality of life and safety.

Of 384 patients enrolled, complete or partial response was achieved in 274 (71%), who were then randomized to MR (n = 140) or RR (n = 134). With a median follow-up of 3.8 years, TTTF was 3.9 years for MR versus 3.6 years for RR (p = not significant). At 3 years, 95% of MR versus 86% of RR patients (p = 0.027) remained free of first cytotoxic therapy, but the mean number of rituximab doses per patient (including the four induction doses) was 15.8 (range 5-31) for MR and 4.5 (range 4-16) for RR. At 12 months post randomization, there was no discernible difference in health related QOL and anxiety between the two arms.

## Summary

The active surveillance therapy strategy in asymptomatic FL patients is based on the disease’s natural history and on the results of trials conducted many years ago. Such trials included patients with different histologic types of asymptomatic lymphoma (based on classifications that are no longer used). Similarly, the treatments used in the past were less effective and more toxic than those used today.

At present, new low-toxicity therapies that prolong progression-free periods and time to new anti-lymphoma therapy are available, among which rituximab monotherapy (with or without maintenance), radioimmunotherapy or the combination of rituximab with bendamustine are the most relevant. Unfortunately, the impact of current therapies on asymptomatic FL patients is still unclear, as only one specific clinical trial has been conducted in this population.

The results of a recently reported clinical trial [39] show that treatment with single-agent rituximab prolongs progression-free survival rates, time to new treatment and the quality of life, as compared with the active surveillance strategy applied to asymptomatic FL patients. However, we do not know if “progression-free survival” or the “time to next treatment” (the main endpoint of the study) are the best end points to decide which option is better in this patient population. It is obvious that patients treated with rituximab will have a superior response rate and longer time to the next treatment than those who were just observed. The critical point is what is going to happen after the next treatment (first-line treatment for observed patients; second-line for the other patients) and, ultimately, with the overall survival of the patients. Furthermore, there is some concern regarding the fact that patients relapsing after rituximab induction plus 2-year maintenance treatment could be particularly resistant to standard immunochemotherapy. Longer-term follow-up of these results is awaited before this treatment can be recommended as the standard initial therapy.

So, it is time to leave the “watch-and-wait” strategy in newly diagnosed asymptomatic follicular lymphoma patients?. Not yet but we are getting closer through effective and less toxic treatments. In this clinical scenario the initial discussion with the patient is typically a long one. Patients need to understand the entire situation of the disease and the risks and benefits of observation versus treatment. The physician must also look at the entire patient, other comorbidities, the patient's mindset and other factors before a final decision can be made. For patients who want active treatment the administration of four weekly rituximab doses should be considered. However, the administration of rituximab maintenance should be avoided until further data based on longer follow-up periods are available.

## Competing interests

Antonio Rueda MD, PhD has received research grant support from Roche and Celgene. María Casanova MD, Maximino Redondo MD, PhD, Elisabeth Pérez-Ruiz MD and Ángeles Medina-Pérez MD have no significant financial interest to disclose

## Authors’ contributions

Antonio Rueda was responsible for the conception and design of the study, and the acquisition, interpretation and analysis of data. María Casanova was also responsible for the design of the study and carried out analysis of data. María Casanova, Maximino Redondo and Elisabeth Pérez-Ruiz contributed to the interpretation of data. Ángeles Medina-Pérez contributed to the conception and design of the study and interpretation of data. All authors read and approved the final manuscript.

## Pre-publication history

The pre-publication history for this paper can be accessed here:

http://www.biomedcentral.com/1471-2407/12/210/prepub
